# Deregulation of miR-183 promotes melanoma development via lncRNA MALAT1 regulation and ITGB1 signal activation

**DOI:** 10.18632/oncotarget.13862

**Published:** 2016-12-10

**Authors:** Yong Sun, Hongyu Cheng, Guangjun Wang, Guojun Yu, Dawei Zhang, Yibing Wang, Wei Fan, Weixi Yang

**Affiliations:** ^1^ Department of Burn and Plastic Surgery, Huai’an First People's Hospital, Nanjing Medical University, Huai’an, China; ^2^ Department of Anesthesiology, Huai’an First People's Hospital, Nanjing Medical University, Huai’an, China

**Keywords:** miR-183, MALAT1, ITGB1, melanoma, competitive endogenous RNA

## Abstract

Dysregulation of miR-183 has been recently elucidated in several carcinomas. However, the expression patterns and mechanisms of miR-183 involved in malignant melanoma remain unidentified. Here, we found down-regulation of miR-183 in melanoma tissues and cells. Decreased level of miR-183 was relevant to poor overall survival, while miR-183 up-regulation resulted in a marked suppression of cell growth *in vitro* and *in vivo*. We further found that the expression and function of miR-183 were suppressed by MALAT1. Integrin β1 (ITGB1) was then speculated and confirmed as a direct target of miR-183. We also illustrated that MALAT1 may function as a sponge competitive endogenous RNA (ceRNA) for miR-183, and thus regulate the molecular expression of ITGB1. Collectively, we found a new signaling pathway promoting melanoma development by MALAT1-miR-183-ITGB1 axis, which may be clinically valuable as new targets for malignant melanoma therapy.

## INTRODUCTION

The incidence of malignant melanoma has showed an increasing trend year by year in the world [1]. Although new targeted treatments and immunotherapy offer higher patient response rates, the long-term survival rates have remained very low, there is therefore an urgent need to comprehend the potential molecular mechanisms related to melanoma process. Moreover, new bio-markers and excise targeted therapies are crucial for the management of melanoma [2–3].

The expression of microRNAs (miRNA) and their functions in carcinogenesis have been extensively elucidated. Generally speaking, miRNAs negatively modulate gene expression at the post-transcriptional level through directly binding to the 3′-untranslated region (3′UTR) of target genes [4]. Abnormal miRNA expression has been frequently observed in various human cancers, including melanoma, indicative of critical roles in proliferation, angiogenesis, drug resistance and metastasis [5–6]. Previous study has shown that miR-183 may exert its effects by directly binding to and inhibiting the promoter activity of integrin β1 (ITGB1), thus imposing a negative regulatory role on cell invasiveness [7]. The investigation of the expression pattern and mechanisms of miR-183 in melanoma remains a large unmet need.

Long non coding RNAs (lncRNAs, >200 nucleotides) have recently gained significant attention in regulating gene expression underlying multiple mechanisms such as epigenetic modulation, decoy, guide and scaffolds [8–9]. The lncRNAs-related regulatory roles are more sophisticated than that of miRNAs. Recently, a novel regulatory model which called competitive endogenous RNA (ceRNA) hypothesis has been largely elaborated between lncRNAs and mRNAs. This model illstruated that mRNA, lncRNA and miRNA could crosstalk with each other. The media of this crosstalk is microRNA binding spot, which also called miRNA response elements (MREs) [10–11]. In previous studies, HOTAIR has been described to be a “ceRNA” that prevents HER2 (mRNA) from miR-331 (miRNA)-mediated expression inhibition [12]. We thus speculate that some other lncRNAs may serve as ceRNAs, connecting miR-183 and the posttranscriptional level system in melanoma.

In order to understand the crosstalk of miRNAs and lncRNAs in human malignant melanoma, we first identified the expression of miR-183 in melanoma and its functional association with tumor growth and overall survival. We then explored that MALAT1 may serve as “sponge ceRNA” to adjust the expression of ITGB1 and tumor growth through inhibiting the expression of miR-183 in melanoma cells, providing a new insight in the therapy of malignant melanoma. In brief, our results revealed a novel regulatory mechanism that was comprised of the MALAT1-miR-183-ITGB1 axis in melanoma.

## RESULTS

### miR-183 was down-regulated in human malignant melanoma and associated with poor overall survival

**Figure 1:**
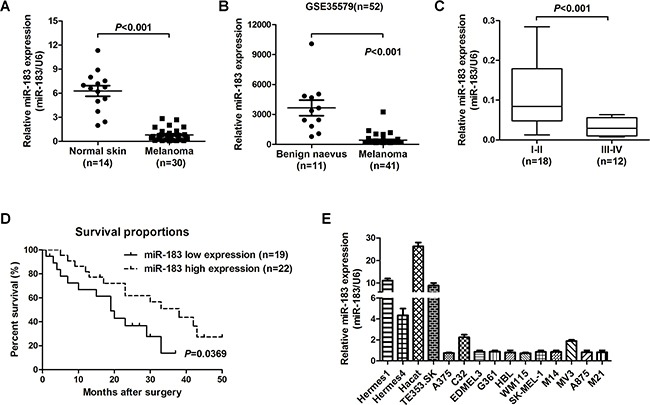
miR-183 was down-regulated in human malignant melanoma and associated with poor overall survival **A**. The levels of miR-183 between normal skin and malignant melanoma tissues were measured using qRT-PCR, U6 RNA is the standardization. **B**. Reanalysis of miR-183 expression from previously published dataset GSE35579 (n=52). **C**. miR-183 expression was measured by qRT-PCR in different advanced pathological stages I-II and stages III-IV. **D**. Kaplan-Meier analysis was used to analysis the overall survival for patients with melanoma according to miR-183 level. **E**. The miR-183 levels were analyzed in various melanoma cell lines and non-cancer cells by qRT-PCR, and U6 was control.

In order to explore miR-183 levels in melanoma tumorigenesis, we analyzed the miR-183 level in 14 normal skin tissues and 30 melanoma tissues by qRT-PCR and found that the miR-183 level in cancer tissues was significantly down-regulated compared with those in normal skin samples (*P* < 0.001, Figure 1A). Re-analyzed a previously published dataset (GEO# GSE 35579), we found the same result (*P* < 0.001, Figure 1B). Examination of the reciprocity between miR-183 expression and clinical pathological features revealed that miR-183 down-regulation was related with advanced pathological stage (Figure 1C). At the mention of overall survival, low miR-183 expression sufferers had a poorer prognosis than those with high miR-183 expression (*P* = 0.0343, Log-rank (Mantel-Cox); Figure 1D). A panel of melanoma cell lines were selected to examine the miR-183 levels. The miR-183 expression was also down-regulated in melanoma cancer cell lines (Figure 1E). These results indicated that low miR-183 expression may be associated with the development of malignant melanoma and poor overall survival.

### miR-183 inhibited melanoma cell proliferation *in vitro* and *vivo*

**Figure 2:**
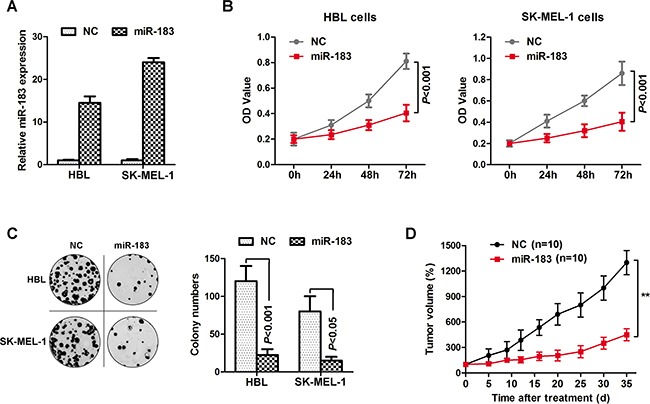
miR-183 inhibited melanoma cell growth **A**. HBL and SK-MEL-1 cells were respectively transfected with miR-183 mimics or negative control, and the expression of miR-183 was validated. **B**. Cell proliferation was detected at 0h, 24h, 48h and 72h by MTS assay, at 490 nm in the average absorbance (OD value). **C**. We performed colony-forming growth assay to test the function of miR-183 mimics, which was respectively transfected to HBL and SK-MEL-1 cells. Colony numbers were then counted. **D**. Tumor growth curves were calculated after HBL cells transfected with miR-183 (n=10, mean ± s.d).

Based on the observations above, we choose lower miR-183 expressing HBL and SK-MEL-1 cells to continue the subsequent experiment. HBL and SK-MEL-1 cells were transfected with a miR-183 mimic or control (Figure 2A). As expected, miR-183 mimic significantly decreased the proliferation of HBL and SK-MEL-1 cells (Figure 2B). We further analyzed cancer cell proliferation through colony formation assays in miR-183-treated HBL and SK-MEL-1 cells. As shown in Figure 2C, miR-183 mimic inhibited the growth of HBL and SK-MEL-1 cells dramatically. To explore the function of miR-183 on tumor development *in vivo*, HBL cells transduced with the miR-183 mimic or control were used in nude mice xenograft models. Up to 35 days, we observed a significant drop in tumor volume in the miR-183 mimic group compared with controls (Figure 2D). These results showed that miR-183 was significantly associated with the growth of melanoma cells, suggesting a potential role of miR-183 as a tumor suppressor in melanoma.

### MALAT1 suppressed miR-183 expression in melanoma

**Figure 3:**
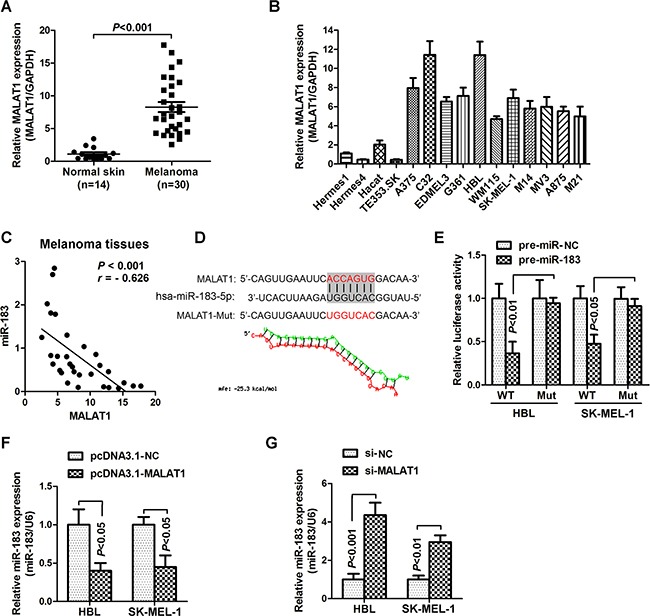
MALAT1 suppressed miR-183 expression in MM **A**. The relative expression of MALAT1 in normal skin (n=14) and melanoma tissues (n=30) was measured using qRT-PCR. **B**. qRT-PCR was performed to measure the level of MALAT1 in various melanoma cells and non-cancer cells. **C**. There was a significant negative correlation between MALAT1 and miR-183 (*r* = -0.626, *P* < 0.001). **D**. Predicted target sites of MALAT1 on miR-183 and the mutant sequence were shown. **E**. The luciferase reporter plasmid which containing mutant (mut) or wild-type (WT) MALAT1 was co-transfected to HBL and SK-MEL-1 cells with pre-miR-183 in parallel with an empty plasmid vector pre-miR-control, and the luciferase activity was measured 48 hour post-transfection. **F**. and **G**. The level of miR-183 was examined after transfected with (F) pcDNA3.1-MALAT1 or (G) si-MALAT1 in HBL and SK-MEL-1 cells.

Emerging evidence indicates that lncRNAs may play a regulatory role as an endogenous RNA ‘sponge’ that influences miRNA expression. Previous studies have demonstrated that MALAT1 (metastasis associated with lung adenocarcinoma transcript-1, also named NEAT2) is over-expressed in multiple carcinomas [14–15], consistent with our findings as it was increased in melanoma cancer tissues and cells (Figure 3A and 3B). Furthermore, we also found a strong negative correlation between MALAT1 and miR-183 expressions (*r* = -0.626, *P* < 0.001) in malignant melanoma tissues (Figure 3C). We then compared the miR-183 with MALAT1 sequence using RNAhybrid and found a miR-83 target site in MALAT1 (Figure 3D). Accordingly, luciferase activity assays were used to examine the suppressive effects of MALAT1 on miR-183 expression. HBL and SK-MEL-1 cells were co-transfected with MALAT1 and miR-183-expressing plasmid. Over-expression of miR-183 dramatically reduced the luciferase activity of cells transfected with wild-type MALAT1 (WT) rather than those with mutant MALAT1 (Mut) (Figure 3E), suggesting that MALAT1 may target miR-183 by binding this putative site. In addition, miR-183 was down-regulated in the HBL and SK-MEL-1 cells transfected with MALAT1 over-expression vector pcDNA3.1-MALAT1 (Figure 3F), while the specific si-MALAT1 significantly up-regulated the miR-183 expression comparing to the negative control (Figure 3G). These results indicated that MALAT1 suppressed miR-183 expression in melanoma.

### MALAT1 regulated tumor growth through miR-183 *in vitro* and *in vivo*

**Figure 4:**
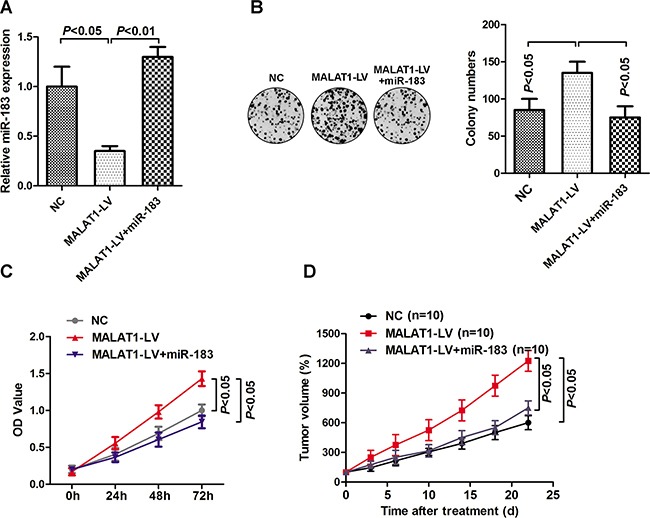
MALAT1 regulated tumor growth through miR-183 **A**. HBL cells were transfected with MALAT1 lentivirus or miR-183 mimic, and the expression of miR-183 was detected. **B**. Cell proliferation was measured by MTS assay, and the OD value was measured at 0h, 24h, 48h and 72h. **C**. Colony formation was detected after cells transfected with MALAT1-LV, MALAT1-LV+miR-183 mimic or control. **D**. Tumor volume was further calculated after injection of HBL cells transfected with MALAT1-LV and miR-183 mimic (n=10).

We further explored the potential roles of MALALT1 and miR-183 on melanoma tumor growth. As shown in Figure 4A, addition of miR-183 mimic significantly restored the inhibitory effect of the MALAT1-LV on miR-183 expression in HBL cells. Enforced expression of MALAT1 significantly promoted cell proliferation and colony formation, which were further inversed by addition of miR-183 mimic (Figure 4B and 4C). Similar results were also observed in *in vivo* studies (Figure 4D). Collectively, these data indicated that MALAT1 promoted melanoma growth by regulating miR-183 expression.

### ITBG1 was verified as a functional target of miR-183

**Figure 5:**
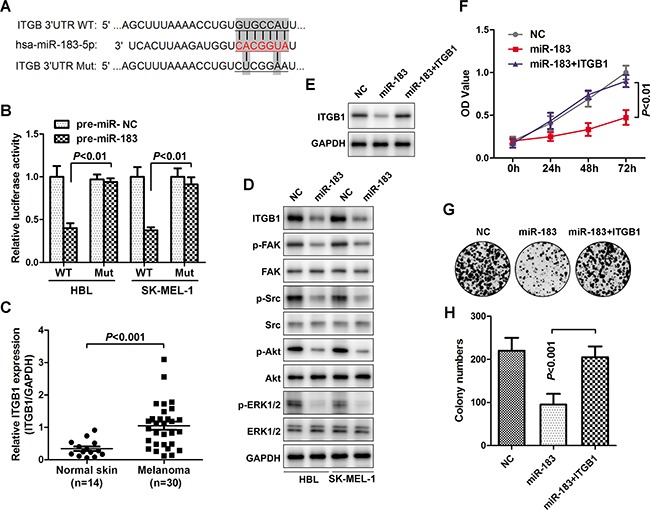
ITBG1 was verified as a functional target of miR-183 **A**. The miRNA target algorithms predicted that the ITGB1 3’-UTR and miR-183 had a conserved binding site, and the wild-type or mutated ITGB1 3’-UTR are designed as above. **B**. Luciferase reporter assay for confirming directly binding of miR-183 to ITGB1 3’-UTR, vector was jointly transfected into HBL or SK-MEL-1 cells together with pre-miR-183, and the luciferase ability was calculated after 48 h. **C**. The relative level of ITGB1 in normal skin (n=14) and melanoma tissues (n=30) was measured using qRT-PCR, normalized to GAPDH. **D**. Western blot determined major ITGB1-associated signaling changes after transfection with miR-183 in HBL and SK-MEL-1 cells. **E**. ITGB1 expression was examined after cells transfected with miR-183 mimic and pcDNA3.1-ITGB1 by Western blot. **F**. The OD value was measured at 0h, 24h, 48h and 72h. **G** and **H**. Colony numbers were further calculated.

The miRNA target algorithms TargetScan (www.targetscan.org) was used to predict the functional target of miR-183. It was found that ITGB1 may be a potential target of miR-183 (Figure 5A). To verify this hypothesis, the 3’UTR of ITGB1 and pre-miR-183 were co-transfected into HBL and SK-MEL-1 cells. Indeed, miR-183 repressed the luciferase activity of wild type 3’UTR of ITGB1 rather than the mutant one. (Figure 5B), suggesting the binding of miR-183 on the 3’UTR of ITGB1. Additionally, ITGB1 was over-expressed in melanoma tissues (Figure 5C), indicating that ITGB may be involved in melanoma development. We further explored the effects of miR-183 on the ITGB1 expression and its downstream signalling events in melanoma. Interestingly, a substantial down regulation of ITGB1, p-FAK, p-Src, p-Akt and p-ERK1/2 were found in miR-183-treated cells (Figure 5D), suggesting the suppression of miR-183 on the ITGB pathway. Furthermore, enforced expression of ITGB1 significantly restored the inhibition of the miR-183 on cell growth and colony formation in melanoma cells (Figure 5E-5H).

### MALAT1 up-regulated ITGB1 expression through sponging miR-183

**Figure 6:**
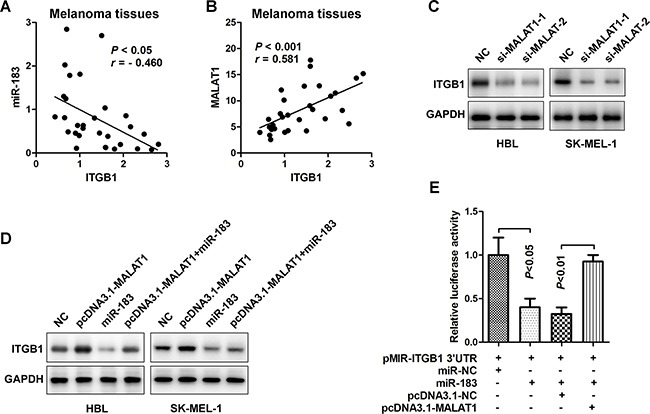
MALAT1 up-regulated ITGB1 expression through sponging miR-183 **A**. The miR-183 and ITGB1 levels appeared a negative correlation (*r* = - 0.460, *P* < 0.05) (n=30). **B**. The expression of MALAT1 in melanoma tissues was positive related to the level of ITGB1 (*r* = 0.581, *P* < 0.001). **C**. Protein expression of ITGB1 in HBL and SK-MEL-1 cells was measured after transfected with si-MALAT1-1 or si-MALAT1-2. **D**. Effects of pcDNA3.1- MALAT1, miR-183, pcDNA3.1-MALAT1+miR-183 on ITGB1 protein in HBL and SK-MEL-1 cells were confirmed using Western blot. **E**. The 3’-UTR of ITGB1 was mixed into the luciferase coding region (pMIR-ITGB1 3’-UTR) and then co-transfected into HEK293T cells with miR-183 or pCDNA3.1-MALAT1. miR-NC and pcDNA3.1-NC were used as control.

**Figure 7:**
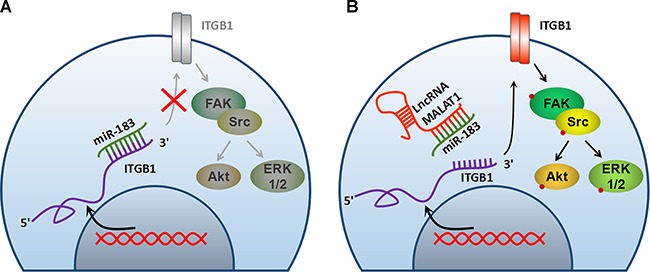
Schematic elaboration of the potential molecular mechanism between MALAT1 and miR-183

Previous study has found that miR-183 can directly regulate ITGB1 expression and promote cell invasion and migration capacities [7, 16]. Consistently, it was demonstrated that miR-183 levels were inversely associated with ITGB1 in this study (Figure 6A, r = - 0.46, *P* < 0.05). We also found an intense positive correlation between MALAT1 and ITGB1 levels (*r* = 0.581, *P* < 0.001) in melanoma tissues (Figure 6B). In order to further verify the regulation of MALAT1 on ITGB1 expression, we examined the protein level of ITGB1 after indicated treatments. As demonstrated in Figure 6C, decreased expression of lncRNA MALAT1 by either si-MALAT1-1 or si-MALAT1-2 repressed ITGB1 protein expression, while up-regulation of MALAT1 by transfecting pcDNA3.1-MALAT1 resulted in the augment of ITGB1 protein level. Melanoma cells with high levels of MALAT1 showed an increase in ITGB1 expression, which could be markedly inversed by transfection with miR-183 (Figure 6D). Furthermore, luciferase assays showed that up-regulating endogenous miR-183 repressed the luciferase activity of Luc-pMIR-ITGB1-3’-UTR, and over-expression of MALAT1 could partially restored such events (Figure 6E). Collectively, these data indicated that MALAT1 may act as an endogenous ‘ceRNA’ through binding to miR-183, thus eliminating the miR183-mediated inhibitory effects on the ITGB1 expression and its downstream signalling events in melanoma (Figure 7).

## DISCUSSION

Malignant melanoma patients in the clinic have limited therapeutic options at present. Hence, it is necessary to explore novel targets for melanoma therapy. In this research, we found that the expression of miR-183 was decreased in melanoma and associated with poor overall survival (Figure 1). In addition, miR-183 significantly decreased melanoma cell proliferation *in vitro* and *in vivo* (Figure 2). Previous studies have suggested that miR-183 may impose a negative regulatory impact on cell invasiveness by directly binding to ITGB1 promoter [7]. Nevertheless, the overall biological function and potential molecular mechanism of miR-183 in melanoma still need to be elucidated.

Growing evidences from studies have elaborated that lncRNAs are vital factors in carcinoma. It has been suggested that RNAs may cross-talk with each other through fighting for the miRNAs response elements (MRE) [17], the endogenous lncRNA could regulate the miRNA post-transcriptionally by acting as competing endogenous RNAs (ceRNAs) [18–19]. For example, lncRNA CCAT1 could act as ceRNA of miRNA-218-5p to correlate with BMI1 mRNA levels in gallbladder cancer [20]. Another case is elucidated by H19, which can derepress the expression and functions of let-7's [21]. Similar reports suggested that there was a mutual restrainment between lncRNA loc285194 and miR-211 in colon carcinoma [22]. However, the excise regulatory mechanisms between lncRNA and miRNA remains to be further elaborated.

The lncRNA MALAT1 has been observed and considered as a pro-oncogene in an expanding list of human tumor tissues or cell lines [23–24]. Here, we identified that MALAT1 was highly expressed in melanoma tissues and cells compared with the non-cancer skin or normal cells (Figure 3). Silencing MALAT1 significantly up-regulated the expression of miR-183, conversely, over-expression of MALAT1 inverted the inhibitory effect of miR-183 on the tumor growth *in vitro* and *in vivo* (Figure 4). Furthermore, we demonstrated a ceRNA activity of MALAT1 by modulating miR-183 in melanoma cells. An increasing number of studies have found the ceRNA activity of lncRNA MALAT1, especially in melanoma. A recent studies have elucidated that MALAT1 down-regulated the expression of tumor-suppressor miR-22 by means of sponging, thus promoted melanoma development [25]. Moreover, the oncogenic role of MALAT1 was reinforced by up-regulating the expression of integrin β1 (ITGB1), a direct target of miR-183. It has been widely reported that over-expression of ITGB1 is associated with tumor growth, metastasis and drug resistance via downstream signaling pathways in various cancers [26–27]. Interestingly, we found a substantial down regulation of ITGB1, p-FAK, p-Src, p-Akt, p-ERK1/2 in miR-183-treated cells (Figure 5D), suggesting a precise regulatory mechanism composed of MALAT1, miR-183, ITGB1 and its downstream signaling pathway, which can be further investigated in future research.. However, we found no significant difference in miR-183 expression between cells derive from primary and metastatic melanoma, consistent with the results obtained from patient tissues (data not shown). It seemed like miR-183 may not be involved in metastasis of melanoma, which need more confirmation.

In general, our study suggested that miR-183 suppressed cell growth by inhibiting ITGB1 signal pathway and MALAT1 promoted melanoma growth by acting as a ceRNA of miR-183 in melanoma. Thus, our findings provided a better understanding of the non-coding RNA interaction regulatory network by MALAT1-miR-183-ITGB1 axis in the development of melanoma.

## MATERIALS AND METHODS

### Tissues and cells

Normal skin and melanoma tissues were obtained with informed consent from participants and approved by the Institutional Review Board (IRB). Some melanoma cancer cell lines (A375, C32, EDMEL3, G361, HBL, WM1115, SK-MEL-1, M14, MV3, A875, M21) and non-cancer cells (Hermes1, Hermes4, Hacat, TE353.SK, HEK293T) were obtained from the ATCC (Manassas, VA, USA). All cancer and non-cancer cells were cultured in RPMI Medium 1640 and Dulbecco's Modifed Eagle's Medium (DMEM) (Hyclone) containing 10% FBS (fetal bovine serum, Hyclone), and were grown in a humidified 5% CO^2^ incubator at 37°C. The miR-183 mimics (5’-UAUGGCACUGGUAGAAUUCACU-3’) and control mimics (5’-UUCUCCGAACGUGUCACGUTT-3’) were synthesised by Genepharma (Shanghai, China).

### RNA extraction and quantitative RT-PCR (qRT-PCR)

Total RNA was extracted from melanoma tissues or cultured cell lines using TRIzol reagent (Invitrogen) according to the instructions. MALAT1 and ITGB1 mRNA expression levels were examined by qRT-PCR. The GAPDH was used as an control. The RNA was then reverse synthesized to cDNA using PrimeScript RT-PCR Kit (Takara). All the real-time RT-PCR reactions were done with SYBR Green Supermix (Bio-Rad, CA, USA). For miR-183, reverse transcription was performed using Applied Biosystems TaqMan MicroRNA Assay. PCR amplifications were started with a 10 min denaturation step at 94°C, followed by 41 amplification cycles (10 s of 94°C, 19 s of 60°C, and 10 s of 71°C).

### Plasmid constructs and transfection assays

MALAT1 or ITGB1 cDNA sequence was synthesized and subcloned into the mammalian expression vector pcDNA3.1 (Invitrogen), with an empty pCDNA3.1 vector as a control. MALAT1 sequence was cloned into the pCDH-CMV-MCS-EF1-copGFP vector, then transfected togrther with fully packaging vector psPAX2 and pMD2.G into 293T cells to amplify MALAT1-lentvirus (MALAT1-LV). SiRNA against MALAT1 (si-MALAT1, Cat. # 4390771) or control (Cat. # 4390843) was obtained from Thermo Fisher Scientific (Lafayette, CO, USA). Cells were transfected using Lipofectamine 3000 Reagent according to the manufacturer's protocol. To construct the luciferase reporter vectors, ITGB1 3’-UTR and MALAT1 cDNA fragment containing the calculated miRNAs binding spots were amplified by PCR, and then subcloned downstream of the luciferase reporter gene in the luciferase vector.

### Colonogenic assay

After transfecation, 300 cells were seeded into six-well plate, and continued to culture for 2 weeks. Crystal violet (0.05%) was used to fix and stain the colonies. And the colony formation assay was performed by counting the number of colone in the six-well plate.

### Western blot assay

For western blot, we firstly extracted and separated the cells protein lysates by SDS-PAGE, and then we transferred the protein to 0.22 µm NC membranes (Sigma) and incubated the specific protein with appointed antibodies (Cell Signaling Technology, Beverly, MA, USA), including ITGB1 (1:1000), p-FAK (1:1000), p-Src (1:1000), p-AKt (1:1000) and p-ERK1/2 (1:1000). Followed by incubation with anti-mouse (1:10000) or anti-rabbit (1:20000) second antibodies (Santa Cruz Biotech), the protein bands were visualized using enhanced chemiluminescence (ECL) reagent (Thermo Scientific, USA).

### Cell growth and viability

Cell growth and viability was analyzed using the 3-(4, 5-Dimethylthiazol-2-yl)-2, 5-diphenyltetra- zolium bromide (MTS, Sigma) assays as previously described [13]. Briefly, 3000 HBL and SK-MEL-1 cells were seeded into 96-well plate after transfection. The cells were maintained for 72 hours and OD value were examined.

### Construction of the dual luciferase reporter assay

The MALAT1 fragment and 3′-UTR of ITGB that contained predicted miR-183 target sites were amplified by PCR. Both mutant and wild-type 3′-UTR fragments were then cloned into pMIR-control vectors. For luciferase reporter assays, wild-type or mutated versions of MALAT1, pMIR-ITGB1-3’UTR, pre-miR-183 or pre-miR-control were transiently transfected into HBL and SK-MEL-1 cells by Lipofectamine 3000 (Invitrgen, USA). Cells were harvested 48 h after transfection, the relative luciferase activities were assayed using the dual-luciferase assay system (Promega) according to the manufacturer's protocol. Transfection was repeated in triplicate.

### *In vivo* animal models

For xenograft models, miR-183 and MALAT1-lentivirus (MALAT1-LV) was constructed and transfected in accordance to the procedure. In total, 5 × 10^6^ transfected cells and their parallel control were subcutaneously injected into the back of 4-week-old female BALB/c mice. The tumor growth was measured with electronic calipers, then the tumor volume was calculated by the equation V (mm^3^) = (Length × Width^2^)/2. All procedures with animal handling were conducted and ratified by the Animal Research Ethics Committee.

### Statistical evaluation

All experiments were performed in triplicate. All data for statistical evaluation were calculated as the means ± s.d. and the results were analyzed using Prism software (GraphPad, CA, USA). Student's *t*-test, One-way ANOVA and Log-rank (Mantel-Cox) test were used to analyze the *in vitro* and *in vivo* data. If *P* < 0.05, we considered there existed statistical significance.
